# The Inflammasome Adaptor Protein ASC in Mild Cognitive Impairment and Alzheimer’s Disease

**DOI:** 10.3390/ijms21134674

**Published:** 2020-06-30

**Authors:** Xavier O. Scott, Marisa E. Stephens, Marie C. Desir, W. Dalton Dietrich, Robert W. Keane, Juan Pablo de Rivero Vaccari

**Affiliations:** 1 Department of Physiology and Biophysics, University of Miami Miller School of Medicine, Miami, FL 33136, USA; x.scott@miami.edu (X.O.S.); rkeane@miami.edu (R.W.K.); 2 Department of Neurological Surgery and The Miami Project to Cure Paralysis, University of Miami Miller School of Medicine, Miami, FL 33136, USA; mes129@miami.edu (M.E.S.); m.desir1@med.miami.edu (M.C.D.); DDietrich@med.miami.edu (W.D.D.); 3 Center for Cognitive Neuroscience and Aging, University of Miami Miller School of Medicine, Miami, FL 33136, USA; 4 InflamaCORE, LLC. Miami, FL 33156, USA

**Keywords:** mild cognitive impairment, Alzheimer’s disease, serum biomarkers, inflammaging, inflammasome, ASC, neurodegeneration

## Abstract

Mild cognitive impairment (MCI) is characterized by memory loss in the absence of dementia and is considered the translational stage between normal aging and early Alzheimer’s disease (AD). Patients with MCI have a greater risk of advancing to AD. Thus, identifying early markers of MCI has the potential to increase the therapeutic window to treat and manage the disease. Protein levels of the inflammasome signaling proteins apoptosis-associated speck-like protein containing a caspase recruitment domain (ASC) and interleukin (IL)-18 were analyzed in the serum of patients with MCI, AD and healthy age-matched donors as possible biomarkers, as well as levels of soluble amyloid precursor proteins α/β (sAPP α/β) and neurofilament light (NfL). Cut-off points and positive and negative predictive values, as well as receiver operator characteristic (ROC) curves, likelihood ratios and accuracy were determined for these proteins. Although the levels of ASC were higher in MCI and AD than in age-matched controls, protein levels of ASC were higher in MCI than in AD cases. For control vs. MCI, the area under the curve (AUC) for ASC was 0.974, with a cut-off point of 264.9 pg/mL. These data were comparable to the AUC for sAPP α and β of 0.9687 and 0.9068, respectively, as well as 0.7734 for NfL. Moreover, similar results were obtained for control vs. AD and MCI vs. AD. These results indicate that ASC is a promising biomarker of MCI and AD.

## 1. Introduction

Alzheimer’s disease (AD) is a neurodegenerative disorder characterized by cognitive and memory decline that worsens over time [1]. The transitional stage between normal aging and early AD is referred to as mild cognitive impairment (MCI) [2]. MCI is characterized by memory impairment in the absence of dementia [3]. Patients with MCI are at greater risk of progressing to AD [4,5]. As a result, it is imperative to identify early markers of MCI that may be used in the monitoring and diagnosis of these patients prior to the development of AD. It is possible that early identification of AD, potentially at the MCI stage, has the benefit to increase therapeutic efficacy when compared to more advanced AD cases.

Cerebrospinal fluid (CSF) amyloid-β (Aβ _(1-42)_) is a promising biomarker for MCI and AD with a specificity and sensitivity above 80% [6]. Aβ _(1-42)_ is present at moderately low levels in cases of Lewy body dementia [7] and decreased in vascular dementia [8]. Similarly, mean Total (T)-tau levels are three times higher in the CSF of AD patients than in controls, with a sensitivity of 82% and a specificity of 88% [9]. Moreover, patients with MCI that develop AD present with high levels of T-tau, with 90% sensitivity and 100% specificity [10]. In addition, soluble amyloid precursor proteins (sAPP) α and β have been shown to be potentially effective biomarkers that may be used in the care of patients with MCI and AD [11].

Neurofilament light chain (NfL) is a cytoskeletal protein and biomarker of axonal damage. NfL levels in the blood (serum and plasma) are correlated with CSF NfL levels [12]. Additionally, plasma NfL levels were shown to be positively correlated with raised cortical microglial activity in brain regions commonly associated with MCI and AD [13]. Moreover, higher plasma and CSF NfL levels correlate with hypometabolism in brain regions consistent with AD [14]. Thus, it appears that NfL levels, combined with additional neuroinflammatory proteins levels, would increase the accuracy of biomarkers to monitor and treat cognitive decline.

Neuroinflammatory responses have been shown to contribute to the underlying pathology of neurodegenerative diseases like MCI, AD and Parkinson’s disease [15]. An inflammatory response as a result of aging is referred to as inflammaging. It has been previously shown that the inflammasome contributes to the naturally occurring process of inflammaging, which likely represents the initial stages of cognitive impairment and neurodegeneration [16,17,18,19]. The inflammasome is a multiprotein complex responsible for the production of interleukin (IL)-1β and IL-18 upon activation of inflammatory caspase-1 [20]. Inflammasome proteins are secreted and play a role in the spread of the inflammatory response. [21] Therefore, secreted inflammasome proteins are promising biomarkers of inflammation. Accordingly, inflammasome proteins have been shown to serve as biomarkers of brain injury [22], stroke [23], multiple sclerosis [24] and depression [25]. Moreover, the inflammasome adaptor protein apoptosis-associated speck-like protein containing a caspase recruitment domain (ASC) has been shown to have prionoid properties, and after secretion, ASC contributes to the spread of inflammation [21]. In addition, ASC forms an aggregate complex with Aβ, which promotes inflammation and pyroptosis in the microglia of mice. [26,27] Thus, the inflammasome is a key contributor to the inflammatory response in AD pathology.

Here, we extend our previous studies to investigate the potential of inflammasome proteins as biomarkers of MCI and AD. We calculated cut-off points, receiver operator characteristic (ROC) curves with associated sensitivity and specificity calculations for the inflammasome proteins ASC and IL-18. In addition, we performed similar analyses for soluble APPα and β (sAPPα/β) and serum NfL to evaluate whether inflammasome proteins serve as more reliable markers for MCI and AD [11].

## 2. Results

### 2.1. ASC and IL-18 Are Elevated in the Serum of Patients with MCI and AD

**Figure 1:**
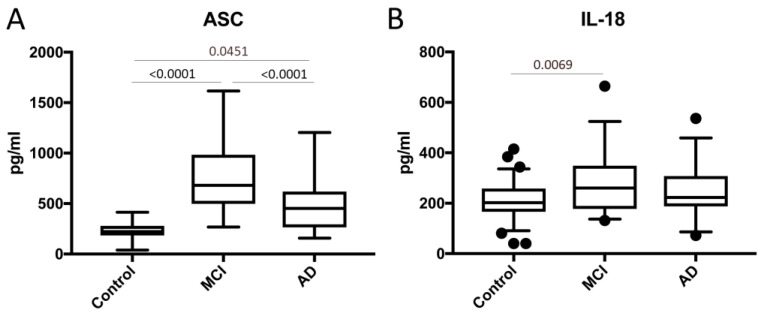
Apoptosis-associated speck-like protein containing a caspase recruitment domain (ASC) and interleukin (IL)-18 are elevated in the serum of patients with mild cognitive impairment (MCI) and Alzheimer’s disease (AD). Protein levels in pg/mL for ASC (**A**) and (**B**) IL-18 in the serum from patients with MCI, AD and controls. ASC: *n* = 66 control, 32 MCI and 31 AD. IL-18: *n* = 69 control, 31 MCI and 32 AD. Box and whiskers are shown for the 5th and 95th percentiles.

To investigate if inflammasome signaling proteins could serve as biomarkers of MCI and AD, we analyzed serum samples from patients with MCI, AD and aged-matched donors for the expression of ASC (Figure 1A) and IL-18 (Figure 1B). Protein levels of ASC and IL-18 were significantly higher in the MCI than in the control group. In addition, serum levels of ASC were found to be significantly higher in MCI than AD patients (Figure 1A). However, there was no statistically significant difference between the serum levels of IL-18 in the MCI vs. AD groups (Figure 1B), suggesting a biomarker role for ASC in the pathology of MCI and AD and IL-18 in MCI.

### 2.2. ASC Is a Promising Serum Biomarker of MCI and AD

To determine if inflammasome signaling proteins may be used as biomarkers of MCI and AD, we determined the area under the curve (AUC) for the control vs. MCI, MCI vs. AD and control vs. AD (Table 1 and Table 2) for ASC and IL-18. For the control vs. MCI, of the inflammasome signaling proteins analyzed, ASC presented the highest AUC of 0.974 (*p* < 0.0001), and IL-18 had an AUC of 0.6896 (*p* = 0.0025) (Table 1); the cut-off point for ASC was 264.9 pg/mL with 100% sensitivity and 74% specificity, whereas IL-18 had a cut-off point of 213.9 pg/mL with 74% sensitivity and 58% specificity (Table 2). For the control vs. AD, the AUC for ASC was 0.8328 (*p* < 0.0001) (Table 1), with a cut-off point of 258.7 pg/mL with 81% sensitivity and 71% specificity (Table 2). Finally, for MCI vs. AD, the AUC for ASC was 0.7157 (*p* = 0.0033) (Table 1), with a cut-off point of < 560 pg/mL and a 71% sensitivity and a 63% specificity (Table 2).

### 2.3. MCI, AD and sAPPα/sAPPβ

**Figure 2:**
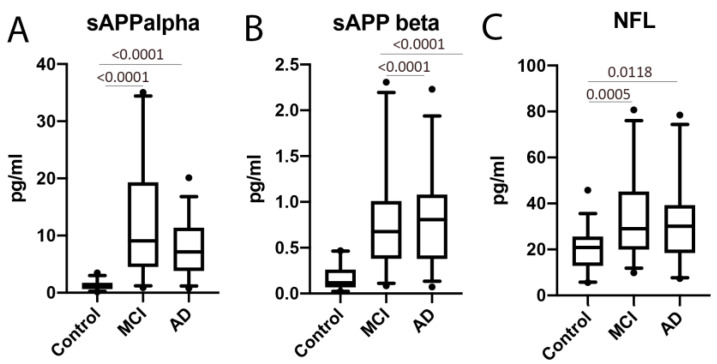
Soluble amyloid precursor proteins (APP) (α and β) and neurofilament light chains were elevated in the serum of MCI and AD patients compared to the control. Protein levels in pg/mL for (**A**) sAPPα, (**B**) sAPPβ and (**C**) neurofilament light (NfL). Soluble (s)APPα: *n* = 35 control, 31 MCI and 32 AD. sAPPβ: *n* = 27 control, 31 MCI and 30 AD. NfL: *n* = 32 control, 32 MCI and 28 AD. Box and whiskers are shown for the 5th and 95th percentiles.

**Figure 3:**
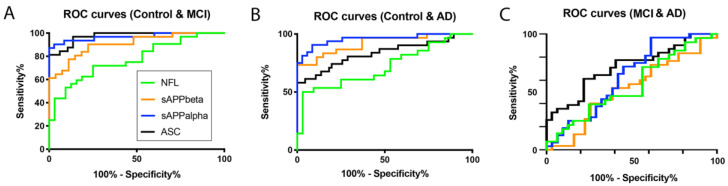
ASC is a promising serum biomarker of MCI. Receiver operator characteristic (ROC) curves for NfL (green), sAPPβ (orange), sAPPα (blue) and ASC (black). (**A**) Control vs. MCI, (**B**) control vs. AD and (**C**) MCI vs. AD.

APP protein levels have been shown to be consistent with the pathology of AD and MCI; thus, it appears that APP is also a promising biomarker for these conditions [11]. Thus, to compare our findings with other more established biomarkers of MCI and AD, we compared the serum protein levels of ASC and sAPPα/sAPPβ for the ability to distinguish between MCI, AD and the controls. In our study, the protein levels of sAPPα (Figure 2A) and sAPPβ (Figure 2B) were higher in MCI and AD patients than in the control subjects. In addition, for the control vs. MCI, the AUC for these two proteins was 0.9687 and 0.9068, respectively (Figure 3 and Table 1), whereas, for the control vs. AD, the AUC were 0.9563 and 0.9185, respectively. In addition, for MCI vs. AD, the AUC were 0.6351 and 0.5247 (Figure 3 and Table 1). For the control vs. MCI, the cut-off point for sAPPα was 1.39 ng/mL and 0.2639 ng/mL for sAPPβ (Table 1). For the control vs. AD, for sAPPα was 2.573 ng/mL and 0.2906 ng/mL for sAPPβ (Table 1). For MCI vs. AD, for sAPPα was 8.846 ng/mL and 0.6364 ng/mL for sAPPβ (Table 1).

In comparison, for the control vs. MCI, the cut-off point for ASC was 264.9 pg/mL with 100% sensitivity and 74% specificity, while sAPPα had a cut-off point of 1.39 ng/mL with 97% sensitivity and 74% specificity, and sAPPβ had a cut-off point of 0.2639 ng/mL with 90% sensitivity and 78% specificity (Table 2).

For the control vs. AD, the cut-off point for ASC was 258.7 pg/mL with 81% sensitivity and 71% specificity, while sAPPα had a cut-off point of 2.573 ng/mL with 91% sensitivity and 91% specificity, and sAPPβ had a cut-off point of 0.2906 ng/mL with 83% sensitivity and 81% specificity (Table 2).

For MCI vs. AD, the cut-off point for ASC was 560.0 pg/mL with 71% sensitivity and 63% specificity, while sAPPα had a cut-off point of 8.846 ng/mL with 72% sensitivity and 55% specificity, and sAPPβ had a cut-off point of 0.6364 ng/mL with 60% sensitivity and 45% specificity (Table 2).

### 2.4. MCI, AD and NfL

Additionally, we compared the serum protein levels of ASC to NfL. When comparing the levels of NfL in the control and MCI patients, we found that the protein levels of NfL were higher in MCI patients than in the control subjects (Figure 2). The AUC for NfL was 0.7734 (Figure 3 and Table 1), whereas, for ASC, it was 0.974, as above stated (Table 1). The cut-off point for NfL was 24.15 pg/mL, with a sensitivity of 72% and a specificity of 75% (Table 2). In comparison, for the control vs. AD, the AUC for NfL was 0.7165, and the cut-off point was 21.48 pg/mL, with 64% sensitivity and 56% specificity (Table 2). However, no significant difference regarding NfL was found between MCI and AD.

### 2.5. Cluster Analysis Using ASC Protein Levels in Control, MCI and AD Patients

**Figure 4:**
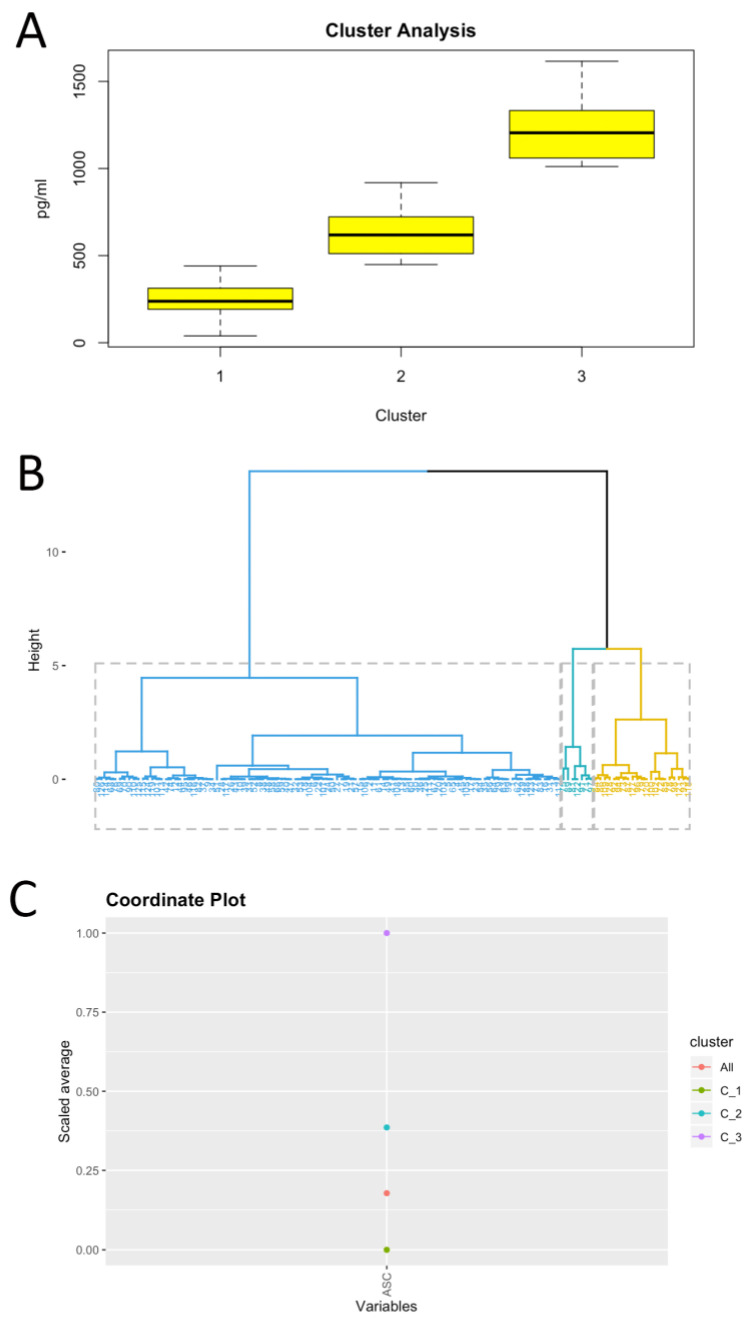
Clustering analysis based on serum ASC protein levels. Clustering using Gaussian Mixture Modeling (**A**), cluster dendrogram using hierarchical clustering (**B**) and coordinate plot (**C**).

Since ASC protein levels are present in the serum of control, MCI and AD patients, we pooled all the concentrations of ASC into one group and performed a cluster analysis. Accordingly, we found three different clusters using a Gaussian Mixture Modeling method (Figure 4A) consistent with the three different cohorts of patients present (control, MCI and AD). In addition, a cluster dendrogram was obtained using hierarchical clustering, in which three groups were also identified (Figure 4B), which was further corroborated in a coordinate plot (Figure 4C). Thus, these findings indicate that ASC protein levels in the serum can be used to stratify patients among the control, MCI and AD cohorts.

## 3. Discussion

In this study, we provide evidence that the inflammasome signaling protein ASC may serve as a biomarker of MCI and AD. ASC and IL-18 were both significantly elevated in the serum of MCI patients when compared to controls, whereas ASC protein levels were also higher in the serum of MCI patients when compared to AD patients. However, there was no statistically significant difference between the levels of IL-18 in AD vs. the control subject. Thus, of all the inflammasome signaling proteins studied (ASC and IL-18), ASC was the protein that presented the best characteristics for a biomarker, including an AUC of 0.974 for the control vs. MCI, 0.8328 for the control vs. AD and 0.7157 for MCI vs. AD.

Importantly, due to the contribution of the inflammasome to inflammaging [17,18,19], we hypothesize that ASC would be a better monitoring biomarker than sAPP α/β and NfL for therapeutic interventions targeting the inflammatory response in patients with MCI. Since there is experimental evidence that sAPPα/β are potential biomarkers for MCI and AD [11], we used these proteins as standards for the comparison of inflammasome proteins as novel biomarkers in these conditions. Our findings in the control vs. MCI groups, that ASC has an AUC of 0.974 compared to 0.9687 for sAPPα, 0.9068 for sAPPβ and 0.7734 for NfL, suggest that ASC is a good biomarker comparable to more traditional biomarkers of MCI, such as sAPP α/β and NfL. Similar results, were found when comparing the control vs. AD. However, when comparing MCI vs. AD, ASC had an AUC of 0.7157, sAPPα 0.6351, sAPPβ 0.5247 and NfL had an AUC of 0.5569, suggesting that, when differentiating MCI vs. AD, ASC is a more reliable serum biomarker.

Interestingly, the levels of ASC were higher in MCI patients than in AD patients. The reason for this decrease in ASC as patients transition from MCI into AD is currently under investigation. However, this is consistent with our previous work on innate immunity and AD, in which retinoic acid-inducible gene-I (RIG-I) expression was also higher in the temporal cortex and plasma of MCI patients [28].

Previous studies have linked inflammation to cognitive impairment [29,30,31,32,33]. A few studies have noted the importance of the C-reactive protein (CRP), a well-studied biomarker of systemic inflammation, to cognitive impairment. High levels of CRP were associated with neuroinflammation and the diagnosis of MCI in patients who had lower scores on executive function and attention tests [32,33,34]. These findings further support the idea that inflammation plays a major role in the development of neurodegeneration in older populations. Recent evidence suggests that the aged brain has a heightened and chronic inflammatory state, which is referred to as inflammaging. Studies in rodents have shown that the activation of the inflammasome contributes to inflammaging of the central nervous system [17,18,19]. Changes in microglial activation have been associated with cognitive impairments in aging nonhuman primates that may contribute to inflammaging [35]. However, although several studies have provided evidence that links inflammation to neurodegeneration, the underlying pathomechanisms attributed to cognitive decline remain to be resolved.

Increased inflammasome protein expression may be associated with other comorbidities that are typical of aging patients. Thus, further studies with better-controlled and stratified patient populations are needed to better characterize inflammasome proteins as biomarkers for the early detection of MCI and AD. In addition, future studies will focus on patients with certain lifestyle parameters that could help stratify different patient populations. For instance, caloric restriction has been shown to modulate genes related to stress and the immune response in the aging brains of mice [36].

We have previously shown that inflammasome proteins can be used as biomarkers of the inflammatory response present in traumatic brain injury [22,37], multiple sclerosis [24], stroke [23] and depression [25]. Here we extend these findings and show that increased inflammasome protein expression occurs in MCI and AD subjects. These findings have important implications for the development of a biomarker panel that may aid physicians in the early detection and diagnosis of MCI and AD. Moreover, the cluster analysis carried in this study indicates that the protein levels of ASC in the serum may be used to stratify patients into three distinct groups—namely, healthy, MCI and AD. Therefore, this study highlights the involvement of ASC in the pathology of MCI and AD. These findings are consistent with animal models that show that inflammasomes, such as the NLRP1 and NLRP3 inflammasomes, play an important role in AD pathology [38,39,40,41].

Moreover, despite ASC being an important marker of the inflammatory component in a variety of diseases or conditions, such as multiple sclerosis, [24] stroke, [23] depression [25] or brain injury [37], how much of an inflammatory component is present in a disease differs among diseases. We believe that the degree in the inflammatory component is marked by the difference in ASC levels between healthy and affected individuals. Thus, the greater the difference, the greater the contribution of ASC to a particular disease. In this study, the difference in ASC protein levels between the control and MCI is greater than between the control and AD. Thus, we hypothesize that ASC is a more significant inflammatory contributor to the pathology of MCI than AD. Moreover, future studies will look at the biomarker role of ASC on other neurodegenerative diseases, such as Parkinson’s disease.

It is important to consider that samples used in this study came from patients that presented several comorbidities that could also contribute to the heightened inflammatory response presented here. Thus, in future studies, we will analyze patients with less comorbidities that could interfere with the analysis. However, despite the comorbidities, since this study was powered for a prevalence of AD and MCI and not the comorbidities, we were able to identify a difference between MCI and AD patients. Furthermore, we carried a binomial logistic regression model to test the odds of patients having MCI or AD based on the levels of ASC, and we determined that the odds of patients having MCI (estimate: 0.022628, *p* = 0.000105) or AD (estimate: 0.011559, *p* = 1.28 × 10^−5^) increased with higher levels of ASC in the serum, thus supporting the involvement of ASC in MCI and AD in this study, regardless of the comorbidities that patients presented. In addition, due to the challenges of diagnosing patients with AD, it is possible that some patients identified as MCI may actually have had AD. Thus, future studies will use better-stratified patients and will aim to correlate the levels of inflammasome proteins with disease severity. These studies will also include a more thorough characterization of the cohort of patients using a battery of neuropsychological tests, as well as magnetic resonance imaging, where the degree of brain atrophy can be quantified and then correlated with the protein levels of ASC. In addition, we will measure the protein levels of ASC, Aβ, T-tau and phospho-tau in the CSF to better understand the relationship between ASC and Aβ, since these two proteins form a complex that is detrimental in mice [26,27]. Furthermore, we will determine whether isolated ASC specks are better biomarkers of AD pathology.

We have previously shown that, in healthy individuals, ASC and IL-18 are higher in the serum of humans over the age of 45 years old. In addition, we have shown that the levels of ASC were higher in Caucasians than in Blacks and Hispanics. Moreover, the protein levels of IL-18 were also higher in Caucasians than in Blacks [19]. In the present investigation, similar numbers of males and females were used for all groups, and the way race was reported in the MCI group did not allow for comparison between races and the diagnoses of MCI/AD. In this study, all patients were above the age of 50 in the control and MCI groups and 47 in the AD group. Thus, since no changes in the serums of patients above the age of 45 have been found for ASC and IL-18 in healthy subjects, we do not anticipate that the effects seen in this study are the result of age, but are, instead, due to the results of MCI/AD pathology. However, logistic regression analyses that included age and the levels of ASC in the serum indicated that, as age and ASC levels increased, so did the odds of developing MCI and AD (data not shown). Therefore, since AD tends to occur later in life than MCI, current studies are looking into the effects of these proteins, with careful standards for patient selection, including patients with MCI and AD that are in the same age range and patients with AD in which the disease severity is known. Unfortunately, in this study, the samples available were not characterized with regards to disease severity but based on physicians’ diagnoses of AD.

Importantly, it has been shown that ASC fibrils bind to Aβ and that microglia recognize this ASC/Aβ complex, resulting in pyroptotic cell death. In addition, extracellular ASC specks may be taken up by microglia, leading to NLRP3 inflammasome activation [26]. Thus, it is possible that the levels of ASC present in the serum play a key role in the pathology of AD by binding to Aβ, further contributing to the deleterious effects associated with neuroinflammation in AD. In addition, the neutralization of ASC with a monoclonal antibody resulted in decreased Aβ pathology in a mouse model of AD [27], an approach that previously has been shown to improve outcomes following CNS injuries [42,43,44,45,46,47]. Thus, current studies are underway to identify which form(s) of ASC (monomer, ASC fibril and ASC speck) is present in the serum of MCI and AD patients and to determine if any of these ASC forms are bound to Aβ. In summary, our study demonstrates that ASC may be used to differentiate inflammation linked to MCI and AD from age-associated inflammation in the elderly population and show that ASC is a promising biomarker for the diagnosis of these neurodegenerative conditions.

## 4. Materials and Methods

### 4.1. Participants

Samples were purchased from BioIVT (Hicksville, NY, USA). Sample donors were enrolled in the study Prospective Collection of Samples for Research sponsored by SeraTrials, LLC, with IRB number 20170439. Samples were obtained after informed consent. Here, we analyzed serum samples from 72 normal male (36 donors) and female (36) donors in the age range of 50 to 70. Normal donors presented no comorbidities. In addition, we analyzed the serum from 32 male (16 donors) and female (16) patients diagnosed with MCI in the age range of 50 to 91 (Table 3) and with AD (22 males and 10 females) in the age range of 47 to 87 (Table 4). In the control group, 20 patients were Caucasian, 32 were Black, 19 were Hispanic and 1 was reported as Other. In the MCI group, 31 patients were reported as Caucasian, and 1 was reported as Caucasian/Japanese. In the MCI group, no distinction or stratification was done between Hispanics and Caucasians. In the AD group, 20 patients were Caucasian, 8 were Black and 4 were Hispanic (some Hispanics were also reported as Caucasians). Patients were classified according to their Atherosclerosis Risk in Communities magnetic resonance imaging (ARIC MRI) cognitive function scores. The scale was developed as part of the Atherosclerosis Risk in Communities (ARIC) study that recruited middle-aged individuals who underwent magnetic resonance imaging (MRI) to evaluate the risk factors of vascular problems in these individuals [48]. Cognitive testing was evaluated using the Delayed Word Recall Test, the Digit Symbol Subtest of the Wechsler Adult Intelligence Scale-Revised (WAIS-R) test and the Controlled Oral Word Association (or Word Fluency) Test of the Multilingual Aphasia Examination [49].

### 4.2. Simple Plex Assay

Analysis of inflammasome proteins (ASC and IL-18) and NfL protein concentration in the serum samples from MCI, AD and controls were performed using the Ella System (Protein Simple, San Jose, CA, USA), as described in [24,50].

### 4.3. MSD Multi-SPOT sAPPα/sAPPβ Assay

Protein levels of soluble APPα and β (sAPPα/sAPPβ) were measured using the MSD 96-well Multi-Spot sAPPα/sAPPβ Assay according to the manufacturer’s instructions (Meso Scale Discovery, Rockville, MD, USA) and read on the MESO Quickplex SQ 120 instrument (Meso Scale Discovery, Rockville, MD, USA). Briefly, the plate was coated with Blocker A solution prior to adding the samples and calibrators, followed by addition of the detection antibody and, ultimately, the reading of the plate in the MESO Quickplex SQ 120 instrument.

### 4.4. Biomarker Analyses

Data obtained by the Simple Plex assay were analyzed with Prism 8 software (GraphPad Software, San Diego, CA, USA). First, outliers were removed, and receiver operating characteristics (ROC) were calculated, thus obtaining a 95% confidence interval, a standard deviation and a p-value. A cut-off point was then obtained for a range of different specificities and sensitivities and their respective likelihood ratio, as well as positive (PPV) and negative predictive values (NPV) and accuracy [24,50].

### 4.5. Statistical Analyses

Normality was tested by the Shapiro-Wilk normality test, and statistical difference between groups was tested by a one-way ANOVA, followed by a Kruskal-Wallis test for multiple comparisons. *p*-value of significance was considered at less than 0.05. In addition, clustering was carried out using hierarchical clustering and Gaussian Mixture Modeling using RStudio software with the following libraries: cluster, caret, factorextra, magrittr, ggplot2 and mclust. Binomial logistic regressions for the outcome of having either MCI or AD were carried out using RStudio software with the following libraries: ggplot2, MASS, dplyr, broom and car.

## Figures and Tables

**Table 1:** Area under the curve. MCI: mild cognitive impairment, AD: Alzheimer’s disease, IL: interleukin, sAPP: soluble amyloid precursor proteins and NfL: neurofilament light.

Biomarker	Area	Std. Error	95% C.I.	*p*-Value
**Control vs. MCI**				
**ASC**	0.974	0.01301	0.9485 to 0.9995	<0.0001
**IL-18**	0.6896	0.06086	0.5703 to 0.8089	0.0025
**sAPPα**	0.9687	0.0216	0.9263 to 1.011	<0.0001
**sAPPβ**	0.9068	0.03784	0.8327 to 0.981	<0.0001
**NfL**	0.7734	0.05821	0.6594 to 0.8875	0.0002
**Control vs. AD**				
**ASC**	0.8328	0.05053	0.7338 to 0.9319	<0.0001
**IL-18**	0.6105	0.06124	0.4905 to 0.7305	0.0749
**sAPPα**	0.9563	0.02490	0.9074 to 1.005	<0.0001
**sAPPβ**	0.9185	0.03592	0.8481 to 0.9889	<0.0001
**NfL**	0.7165	0.06817	0.5829 to 0.8501	0.0040
**MCI vs. AD**				
**ASC**	0.7157	0.06472	0.5889 to 0.8426	0.0033
**IL-18**	0.5847	0.07332	0.441 to 0.7284	0.2482
**sAPPα**	0.6351	0.07146	0.4950 to 0.7752	0.0654
**sAPPβ**	0.5247	0.07514	0.3774 to 0.6720	0.7401
**NfL**	0.5569	0.07502	0.4099 to 0.7040	0.4498

**Table 2:** Sensitivity and specificity. PPV: positive predictive values and NPV: negative predictive values.

Biomarker	Cut-Off Point (pg/mL)	Sensitivity (%)	Specificity (%)	PPV (%)	NPV (%)	Likelihood Ratio	Accuracy (%)
**Control vs. MCI**							
**ASC**	>264.9	100	74	65	100	3.882	83
**IL-18**	>213.9	74	58	44	83	1.765	63
**sAPPα**	>1.39 (ng/mL)	97	74	81	95	3.763	86
**sAPPβ**	>0.2639 (ng/mL)	90	78	78	90	4.065	84
**NfL**	>24.15	72	75	71	75	2.875	74
**Control vs. AD**							
**ASC**	>258.7	81	71	57	89	2.801	74
**IL-18**	>196.5	72	42	37	76	1.24	51
**sAPPα**	>2.573 (ng/mL)	91	91	92	90	10.57	91
**sAPPβ**	>0.2906 (ng/mL)	83	81	80	85	4.5	82
**NfL**	>21.48	64	56	56	64	1.469	60
**MCI vs. AD**							
**ASC**	<560.0	71	63	65	69	1.892	67
**IL-18**	>290.3	72	48	59	63	1.393	60
**sAPPα**	<8.846 (ng/mL)	72	55	64	63	1.592	64
**sAPPβ**	>0.6364 (ng/mL)	60	45	49	56	1.094	52
**NfL**	<33.92	71	44	53	64	1.27	57

**Table 3:** Patients with MCI used in the study. ARIC MRI: Atherosclerosis Risk in Communities magnetic resonance imaging.

Age	Gender	Race	Diagnosis	Medications	Historical Test
83	Male	Caucasian	Mild Cognitive Impairment (MCI), Prostate Cancer, Methicillin Resistant Staphylococcus Aureus Infection, Hyperlipidemia (HLD), Hypertension (HTN), Diverticulitis, Amnesia	Omega 3 1000 mg, Plavix 75 mg, Toprol 50 mg, Vitamin B12-Folic Acid 0.5–1 mg, Vitamin D 400 IU, Zetia 10 mg	ARIC MRI Cognitive Function Score = 18 (20 February 2018)
81	Female	Caucasian	Mild Cognitive Impairment (MCI), Type 2 Diabetes, Hypercholesterolemia	Aspirin 81 mg, Gabapentin 100 m, Eliquis 2.5 mg, Ranitidine 150 mg, Aricept 10 mg	ARIC MRI Cognitive Function Score = 18 (22 May 2018)
62	Male	Caucasian	Mild Cognitive Impairment (MCI), Type 2 Diabetes, Hypertension (HTN), Hyperlipidemia (HLD), Asthma	Omeprazole 20 mg, Benicar 40–12.5 mg, Metformin HCLl 500 mg, Glucotrol XL 5 mg, Singulair 10 mg, Clobetasol Propionate 0.05%, Glipizide 5 mg, Advair Diskus 250/50 μg, Crestor 10 mg, Ipratropium-Albuterol 0.5–2.5 mg/3 mL, Ventolin HFA 108 μg	ARIC MRI Cognitive Function Score = 30 (15 May 2018)
69	Female	Caucasian	Mild Cognitive Impairment (MCI), Asthma, Chronic Obstructive Pulmonary Disease (COPD), Hypertension (HTN)	Alendronate 70 mg, Meclizine 12.5 mg, Prozac 40 mg, Seroquel 50 mg, Trilipix 54 mg	ARIC MRI Cognitive Function Score = 21 (30 May 2018)
75	Male	Caucasian	Mild Cognitive Impairment (MCI), Colon Cancer	Vitamin B12 2500 IU, Avastin, Adrucil, Amoxicillin 500 mg, Lisinopril 20 mg, Metformin HCLl 500 mg	ARIC MRI Cognitive Function Score = 12 (27 March 2018)
72	Male	Caucasian	Mild Cognitive Impairment (MCI), Benign Prostatic Hyperplasia (BPH), Lumbar Spondylosis, Barrett’s Esophagus, Atrial Ectopy, Hypertension (HTN)	Tamsulosin HCLl 0.4 mg, Finasteride 5 mg, Multivitamin, Fish Oil 1000 mg, Viagra 100 mg, Tramadol HCLl 50 mg	ARIC MRI Cognitive Function Score = 15 (10 May 2018)
64	Male	Caucasian	Mild Cognitive Impairment (MCI), Type 2 Diabetes, Hypertension (HTN), Hypercholesterolemia, Benign Prostatic Hyperplasia (BPH)	Zolpidem 10 mg, Cialis 5 mg, Aspirin 81 mg, Tamsulosin 0.4 mg, Rosuvastatin 20 mg, Metformin 500 mg	ARIC MRI Cognitive Function Score = 34 (4 April 2018)
84	Female	Caucasian	Mild Cognitive Impairment (MCI), Hypertension (HTN), Psychoses, Cellulitis, Mitral Valve Prolapse (MVP), Hyperlipidemia (HLD)	Simvastatin 20 mg, Potassium Chloride 10 mEq, Amlodipine Besylate 2.5 mg, Dutasteride 0.5 mg, Losartan Potassium 100 mg, Aspirin 81 mg, Furosemide 20 mg, Potassium Chloride 10 mEq, Avodart 0.4 mg, Amlodipine Besylate 2.5 mg, Ramipril 10 mg	ARIC MRI Cognitive Function Score = 8 (10 May 2018)
68	Female	Caucasian	Mild Cognitive Impairment (MCI), Multiple Sclerosis	Tysabri, Lexapro, Gabapentin	ARIC MRI Cognitive Function Score = 15 (6 April 2018)
69	Female	Caucasian	Mild Cognitive Impairment (MCI), Hypercholesterolemia, Hypertension (HTN), Type 2 Diabetes, Premature Ventricular Contraction	Crestor 5 mg, Omega 3, Zolpidem Tartrate 5 mg, Glucosamine 1500 mg, Fiber, Calcium, Multivitamin, Zyrtec, Chlordiazepoxide-Clidinium 2.5–5 mg, Valacyclovir 500 mg, Lisinopril 10 mg, Janumet 50–500 mg, Metoprolol Succinate 25 mg, Levothyroxine Sodium 100 μg, Rosuvastatin Calcium 5 mg, Omega 3-Acid Ethyl Esters 1 g, Trazodone 50 mg	ARIC MRI Cognitive Function Score = 33 (1 May 2018)
50	Female	Caucasian	Mild Cognitive Impairment (MCI), Hypercholesterolemia	None	ARIC MRI Cognitive Function Score = 30 (24 April 2018)
78	Male	Caucasian	Mild Cognitive Impairment (MCI)	Zaleplon 10 mg, Lorazepam 1 mg, Plavix 75 mg, Aspirin, Allopurinol 300 mg, Levothyroxine Sodium 125mcg, Atorvastatin Calcium 20 mg, Metformin HCLl 1000 mg, Pantoprazole Sodium 40 mg	ARIC MRI Cognitive Function Score = 24 (27 April 2018)
77	Male	Caucasian	Mild Cognitive Impairment (MCI), Hypertension (HTN), Hyperlipidemia (HLD), Vitamin D Deficiency	Aciphex 20 mg, Citric Acid-d-Gluconic Acid, Avodart 0.5 mg, Cozaar 100 mg, Ranitidine Acid Reducer 75 mg, Polyethylene Glycol, MiraLAX, Symbicort 4.5–80 μg, Proair 108 μg, Ipratropium Bromide 0.03%, Prevacid 15 mg, Losartan Potassium 100 mg, Levocetirizine Dihydrochloride 5 mg, Cialis 5 mg, Albuterol, Rabeprazole Sodium 20 mg, Atorvastatin Calcium 20 mg	ARIC MRI Cognitive Function Score = 24 (9 May 2018)
73	Female	Caucasian	Mild Cognitive Impairment (MCI), Hypercholesterolemia, Hypothyroidism, Hypothyroidism, Gastroesophageal Reflux Disease (GERD), Vitamin D Deficiency, Hypertension (HTN)	Rabeprazole Sodium 20 mg, Synthroid 75 μg, Crestor 5 mg, Zyrtec Allergy 10 mg, Aspirin, Calcium 150 mg, CoQ10 400 mg, Aciphex 20 mg, Zenpep 3000 IU-10,000 IU, Ipratropium Bromide 0.03%, Rosuvastatin Calcium 5 mg	ARIC MRI Cognitive Function Score = 37 (9 May 2018)
71	Male	Caucasian	Mild Cognitive Impairment (MCI), Dyslipidemia, Valvular Heart Disease, Hypertension (HTN), Hyperlipidemia (HLD), Aortic Aneurysm, Ulcerative Colitis (UC)	Epipen, Metoprolol Succinate ER 50 mg, Zyrtec, Montelukast, Pepcid, Tramadol 50 mg, Diazepam 5 mg, Metamucil 48.57%, Aspirin 81 mg, Plavix 75 mg, Nexium 40 mg, Lipitor 10 mg, Asacol 800 mg	ARIC MRI Cognitive Function Score = 24 (10 May 2018)
74	Female	Caucasian	Mild Cognitive Impairment (MCI), Asthma, Chronic Obstructive Pulmonary Disease (COPD), Type 2 Diabetes, Hypercholesterolemia, Congestive Heart Failure (CHF), Hypothyroidism	Levothyroxine 75 mg, Metformin 500 mg, Losartan 100 mg, Symbicort, Proventil, Calcium, Vitamin D3, Zyrtec 10 mg	ARIC MRI Cognitive Function Score = 30 (11 May 2018)
75	Male	Caucasian	Mild Cognitive Impairment (MCI), Neuropathy, Benign Prostatic Hyperplasia (BPH), Hypertension (HTN), Rheumatoid Arthritis (RA), Sjogren’s Syndrome, Glaucoma, Allergic Rhinitis, Nasal Obstruction, Type 2 Diabetes	Patanase 0.6%, Timolol Hemihydrate, Latanoprost 0.005%, Methotrexate, Prednisone, Folic Acid, Vitamin D, Finasteride 5 mg, Tamsulosin HCLl 0.4 mg, Gabapentin 100 mg, Vicodin 5–300 mg, Losartan Potassium 50 mg, Pilocarpine HCLl 5 mg, Calcium 600 mg, Vitamin B12 100 μg, Docusate Sodium 100 mg, MiraLAX, Polyethylene Glycol, Ventolin HFA 90 μg, Azithromycin 250 mg, Lasix 20 mg, Levaquin 500 mg, Evoxac 30 mg	ARIC MRI Cognitive Function Score = Refused (18 May 2018)
75	Male	Caucasian	Mild Cognitive Impairment (MCI), Hypercholesterolemia, Thyroid Disease	Levothyroxine Sodium 25 μg, Crestor 40 mg	ARIC MRI Cognitive Function Score = 35 (24 May 2018)
75	Male	Caucasian	Mild Cognitive Impairment (MCI), Hypercholesterolemia, Age Related Macular Degeneration (AMD), Erectile Dysfunction (ED)	Pravachol 40 mg, Ocuvite, Viagra 50 mg	ARIC MRI Cognitive Function Score = 31 (19 February 2018)
75	Female	Caucasian	Mild Cognitive Impairment (MCI), Type 2 Diabetes, Hypertension (HTN), Dyslipidemia, Chronic Kidney Disease (CKD), Pulmonary Nodule, Hyperlipidemia (HLD)	Metformin 500 mg, Atorvastatin Calcium 20 mg, Cozaar 100 mg, Aspirin 81 mg, Hydrochlorothiazide 25 mg, Lipitor 20 mg	ARIC MRI Cognitive Function Score = 42 (1 May 2018)
76	Female	Caucasian	Mild Cognitive Impairment (MCI), Hyperlipidemia (HLD), Hypertension (HTN), Gastroesophageal Reflux Disease (GERD), Anxiety, Hypothyroidism	Donepezil HCL 10 mg, Levothyroxine Sodium 50 μg, Tramadol HCLl 50 mg, Atorvastatin Calcium 20 mg, Omeprazole 20 mg, Losartan Potassium 50 mg, Aricept 10 mg, Paxil 20 mg, Namenda 10 mg	ARIC MRI Cognitive Function Score = 7 (4 May 2018)
76	Male	Caucasian	Mild Cognitive Impairment (MCI), Hypertension (HTN), Type 2 Diabetes, Peripheral Polyneuropathy, Benign Prostatic Hyperplasia (BPH)	Novolog, Lantus 100 U/mL, Metoprolol Succinate 25 mg, Tacrolimus, Terazosin HCLL 10 mg, CellCept 250 mg, Aspirin 81 mg, Allopurinol 150 mg, Atorvastatin Calcium 10 mg, Losartan Potassium 100 mg	ARIC MRI Cognitive Function Score = 28 (15 May 2018)
67	Female	Caucasian	Mild Cognitive Impairment (MCI), Asthma, Hypercholesterolemia	Crestor 40 mg, Omeprazole 20 mg	ARIC MRI Cognitive Function Score = 40 (7 May 2018)
56	Female	Caucasian/Japanese	Mild Cognitive Impairment (MCI)	Daily Vitamins, Aspirin 81 mg	ARIC MRI Cognitive Function Score = 41 (8 May 2018)
58	Female	Caucasian	Mild Cognitive Impairment (MCI), Hyperlipidemia (HLD)	Simvastatin 20 mg, Caltrate 600 mg-Vitamin D 800 IU, Vitamin D 2000 IU, Ibuprofen 800 mg, Prolia 60 mg/mL	ARIC MRI Cognitive Function Score = 42 (8 May 2018)
75	Female	Caucasian	Mild Cognitive Impairment (MCI), AF, Dyslipidemia, Hypertension (HTN), Hypothyroidism	Crestor 10 mg, Armour Thyroid 60 mg, Ramipril 5 mg, Hydrochlorothiazide 25 mg, Promethium 200 mg, Augmentin 125–875 mg, Rosuvastatin Calcium 10 mg	ARIC MRI Cognitive Function Score = 31 (11 May 2018)
84	Female	Caucasian	Mild Cognitive Impairment (MCI), Venous Insufficiency, Hyperlipidemia (HLD), Hypothyroidism, Parkinson’s Disease (PD), Mitral Valve Prolapse (MVP), Anxiety	Cipro 500 mg, Ibuprofen 800 mg, Xanax 0.5 mg, Fluconazole 150 mg, Carbidopa-Levodopa 25–100 mg, Potassium Chloride 20 mEq, Simvastatin 20 mg, Furosemide 40 mg, Levothyroxine Sodium 75 μg, Atenolol 25 mg, Lasix, Aspirin 81 mg, Acetaminophen 500 mg	ARIC MRI Cognitive Function Score = 19 (11 May 2018)
88	N/A	Caucasian	Mild Cognitive Impairment (MCI), Hyperlipidemia (HLD), Peripheral Vascular Disease, Hypertension (HTN), Hyperlipidemia, Mild Intermittent Asthma, Hypercholesterolemia, Type 2 Diabetes	Cozaar 100 mg, Crestor 10 mg, Aspirin, Prilosec 20 mg, Amlodipine Besylate 5 mg, D3 1000 IU, Vitamin C 100 mg, Multi for Him, Omeprazole 20 mg	ARIC MRI Cognitive Function Score = 8 (22 May 2018)
71	Male	Caucasian	Mild Cognitive Impairment (MCI), Hypertension (HTN), Hypercholesterolemia, Chronic Kidney Disease (CKD), Palsy of Conjugate Gaze, Short Term Memory, Hyperlipidemia, Cervical Spondylosis, Basal Cell Cancer (BCC), Complex Partial Epileptic Seizure, Chronic Tremor, Lumbosacral Radiculitis, Allergic Rhinitis, Lumbar Arthritis, Arthritis, Bilateral Hearing Loss	Aspirin 81 mg, Brimonidine 0.15%, Cialis 20 mg, Dexamethasone 4 mg/mL, Donepezil 5 mg, Fexofenadine 180 mg, Lamotrigine 200 mg, Lisinopril 5 mg, Meloxicam 15 mg, Pramipexole 0.25 mg, Simvastatin 40 mg, Virtussin 10–100 mg/5 mL	ARIC MRI Cognitive Function Score = 44 (24 May 2018)
86	Male	Caucasian	Mild Cognitive Impairment (MCI), Hypertensive Heart and Renal Disease with Congestive Heart Failure, Cyst and Pseudocyst of Pancreas, Benign Prostatic Hyperplasia (BPH), Type 2 Diabetes, Chronic Kidney Disease (CKD), Hypokalemia, Chronic Systolic Heart Disease, Mitral Valve Prolapse (MVP), Atrial Fibrillation (AF), Hyperlipidemia, Sensorineural Hearing Loss, Left Bundle Branch Block, Pulmonary Hypertension (HTN), Hyperparathyroidism	Amlodipine 5 mg, Glimepiride 1 mg, Nitroglycerin 0.2 mg, Potassium Chloride 20 mEq, Warfarin 2 mg	ARIC MRI Cognitive Function Score = 48 (17 May 2018)
91	Female	Caucasian	Mild Cognitive Impairment (MCI), Type 2 Diabetes, Hypertension (HTN), Hypercholesterolemia, Benign Prostate Hyperplasia (BPH), Abdominal Aortic Aneurysm, Atrial Fibrillation (AF)	Amlodipine Besylate 5 mg, Atorvastatin Calcium 40 mg, Coumadin, Plavix 75 mg, Toprol 50 mg	ARIC MRI Cognitive Function Score = 31 (13 March 2018)
88	Male	Caucasian	Mild Cognitive Impairment (MCI), Hypercholesterolemia, Melanoma, Depression, Squamous Cell Carcinoma, GERD, Hemorrhoids, TIA	Trintellix 10 mg, Aripiprazole 2.5 mg, Rosuvastatin 20 mg, Modafinil 200 mg, Amphetamine 20 mg, Namenda 28 mg, Esomeprazole 20 mg, Lutein 5 mg, Vitamin D3 1000 IU, Aspirin 81 mg, Vitamin B12	ARIC MRI Cognitive Function Score = 16 (21 February 2018)

**Table 4:** Patients with AD used in this study.

Gender	Age	Race	Diagnosis	Medications
Male	82	Caucasian	Alzheimer’s Disease (AD), Gastroesophageal Reflux Disease (GERD), Benign Prostatic Hyperplasia (BPH), Sleep Apnea, Malignant Basal Cell Neoplasm of Skin, Depression, Dermatitis, Osteoarthritis (OA), Thrombocytopenia	Aricept 10 mg, B Complex 100 0.4 mg, Doxazosin 8 mg, Finasteride 5 mg, Melatonin 10 mg, Multivitamin 9 mg, Omeprazole 20 mg, Sertraline, Simvastatin 80 mg, Vitamin D3 2000 IU, Voltaren 1%
Male	87	Caucasian	Alzheimer’s Disease (AD), Hypertension (HTN), Hyperlipidemia, Dementia	Cartia XT 120 mg, Prilosec 20 mg, Namenda 28XL, Exelon Patch 9.5 mg, Paxil 20 mg
Female	84	Caucasian	Hypertension (HTN), Vitamin D Deficiency, Hyperlipidemia (HLD), Skin Cancer, Anemia, Alzheimer’s Disease (AD)	Cerefolin NAC 6–200 mg, Clopidogrel Bisulfate 75 mg, Multivitamin, Galantamine Hydrobromide ER 16 mg, Memantine HCLHCL 10 mg, Vitamin D3, Zolpidem Tartrate 5 mg, Iron 325 mg, Remeron 15 mg, Plavix 75 mg
Female	76	Caucasian	Hyperlipidemia (HLD), Hypertension (HTN), Gastroesophageal Reflux Disease (GERD), Anxiety, Alzheimer’s Disease (AD), Hypothyroidism	Donepezil HCLHCL 10 mg, Levothyroxine Sodium 50 μg, Tramadol HCLHCL50 mg, Atorvastatin Calcium 20 mg, Omeprazole 20 mg, Losartan Potassium 50 mg, Aricept 10 mg, Paxil 20 mg, Namenda 10 mg
Male	47	Caucasian	Alzheimer’s Disease (AD)	Donepezil 10 mg
Male	67	African	Alzheimer’s Disease (AD)	Rivastigmine 3 mg, Multivitamin
Male	61	Caucasian	Alzheimer’s Disease (AD), Type 2 Diabetes, Hypertension (HTN), Hypercholesterolemia	Atorvastatin 40 mg, Gabapentin 300 mg, Aspirin 81 mg, Razadyne 16 mg, Metformin 500 mg
Female	60	African	Alzheimer’s Disease (AD), Hypertension (HTN)	Clonidine 0.3 mg, Ambien, Quetiapine 300 mg
Male	47	N/A	Alzheimer’s Disease (AD), Asthma, Anxiety	Gabapentin 300 mg
Male	60	African	Alzheimer’s Disease (AD), Type 2 Diabetes	Donepezil, Metformin, Humalog
Male	74	Caucasian	Alzheimer’s Disease (AD), Hypertension (HTN), Hypercholesterolemia	Aspirin 80 mg, Plavix 75 mg, Lisinopril 25 mg, Simvastatin 10 mg, Digoxin 30 mg, Metoprolol 50 mg, Razadyne 24 mg
Male	50	African	Alzheimer’s Disease (AD), Seizures	Keppra 500-750 mg, Exelon Patch
Male	67	African	Alzheimer’s Disease (AD), Hypertension (HTN)	Aspirin 81 mg, Lisinopril 5 mg, Metoprolol Succinate 500 mg
Male	59	Mixed Race	Alzheimer’s Disease (AD), Type 2 Diabetes, Hypertension (HTN), Anxiety	Metoprolol 50 mg, Amlodipine/Benazepril 10–40 mg, Seroquel 50 mg, Aricept 23 mg, Creon 36000 IU, Gabapentin 600 mg, Prandin 2 mg, Metformin 1000 mg
Male	54	African	Alzheimer’s Disease (AD), HTN	Donepezil 10 mg, Multivitamin, Atenolol 50 mg
Female	58	N/A	Alzheimer’s Disease (AD), Asthma, Hypertension (HTN), Hypercholesterolemia, Rheumatoid Arthritis (RA), Type 2 Diabetes	Combivent 103 μg, Symbicort 160 μg, Budesonide 0.5 mg, Singulair 10 mg, Prandin 2 mg, Metoprolol 50 mg, Lotrel 20 mg, Janumet 1000 mg, Donepezil 10 mg, Maxzide 37.5 mg
Male	75	Caucasian	Osteomyelitis, Type 2 Diabetes, Chronic Kidney Disease (CKD), Dyslipidemia, Hypertension (HTN), Erectile Dysfunction (ED), Atherosclerosis, Alzheimer’s Disease (AD)	Hydrochlorothiazide 25 mg, Humalog 100 U/mL, Lantus 100 U/mL, Metformin HCLHCL 1000 mg, Testosterone Cypionate 200 mg/mL, Amlodipine Besylate 10 mg, Ventolin HFA 108 μg, Carvedilol 25 mg, Lipitor 20 mg, Benazepril HCLHCL 40 mg, Azithromycin 250 mg, Proair 108 μg
Female	75	Caucasian	Alzheimer’s Disease (AD), Allergy (Seasonal)	Aricept 10 mg, Namenda 10 mg, Calcitrate 200 mg, Centrum Silver, Cetirizine 10 mg, Folic Acid 400 μg, Magnesium 250 mg
Female	73	Caucasian	Alzheimer’s Disease (AD), Type 2 Diabetes, Hypercholesterolemia, Coronary Artery Disease (CAD)	Vitamin D6, Folic Acid, Warfarin 5 mg, Losartan/Hydrochlorothiazide 50 mg/12.5 mg, Metformin 500 mg, Aricept 10 mg
Male	55	N/A	Alzheimer’s Disease (AD), Hypertension (HTN), Bilateral Carpal Tunnel	Losartan/Hydrochlorothiazide 50 mg/12.5 mg, Meloxicam 15 mg, Norvasc 10 mg
Male	84	Caucasian	Hypertension (HTN), Hypercholesterolemia, Alzheimer’s Disease (AD)	Metoprolol 25 mg, Atorvastatin 40 mg, Aspirin 81 mg, Theragran
Male	51	African	Alzheimer’s Disease (AD), Hypertension (HTN), Hypercholesterolemia	Hydrochlorothiazide 25 mg, Razadyne 16 mg
Male	64	N/A	Alzheimer’s Disease (AD), Hypertension (HTN), Hypercholesterolemia, Type 2 Diabetes	Exelon 6 mg, Metformin 500 mg, Atorvastatin 40 μg, Ramipril 10 mg, Lantus 100 U/mL
Female	84	Caucasian	Hypertension (HTN), Hallucinations, Psychoses, Cellulitis, Dementia, Mitral Valve Prolapse (MVP), Hyperlipidemia (HLD), Alzheimer’s Disease (AD)	Simvastatin 20 mg, Potassium Chloride 10 mEq, Amlodipine Besylate 2.5 mg, Dutasteride 0.5 mg, Losartan Potassium 100 mg, Aspirin 81 mg, Furosemide 20 mg, Potassium Chloride 10 mEq, Avodart 0.4 mg, Amlodipine Besylate 2.5 mg, Ramipril 10 mg
Female	62	Caucasian	Sporadic Alzheimer’s Disease (AD), Asthma	Topamax 150 mg, Vesicare 5 mg, Prozac 60 mg, Levoxyl 75 mg, Xarelto 20 mg, Hydrocodone-Acetaminophen 5–325 mg, Butran Patch 15 mg, Gabapentin 600 mg, Celebrex 200 mg, Breo 100 mg, ProAir, Bentyl 20 mg, Pantoprazole 40 mg
Male	68	Caucasian	Alzheimer’s Disease (AD), Type 2 Diabetes, Hypertension (HTN), Hypercholesterolemia, Cerebrovascular Accident (CVA), Parkinsonism, Peripheral Neuropathy, Hypothyroidism, Benign Prostatic Hyperplasia (BPH), Depression, Anxiety, Glaucoma, Hernia	NamEnda 5 mg, Tamsulosin HCLHCL 0.4 mg, Atorvastatin 40 mg, Valsartan 320 mg, Zetia 10 mg, Carvedilol 25 mg, Aspirin 325 mg, Bupropion HCLl ER 200 mg, Venlafaxine ER 150 mg, Finasteride 5 mg, Synthroid 50 μg, Zolpidem 10 mg, Novolog 100 units/mL, Lantus 100 units/mL, Latanoprost 0.005%, Azelastine 0.15%, Glucagon 1 mg
Male	72	Caucasian	Hypertension (HTN), Hypercholesterolemia, Alzheimer’s Disease (AD)	Omega-3 Fatty acids/Docosahexanoic acid/EPA/Fish oil 350 mg/235 mg/90 mg/597 mg, CoQ10 100 mg, Vitamin B Complex, Aspirin 81 mg, Pravastatin 20 mg, Losartan 50 mg, Namenda XR 28 mg, Donepezil 10 mg, Crenizumab
Male	79	Caucasian	Asthma, Hypertension (HTN), Hypercholesterolemia, Basal Cell Cancer (BCC), Alzheimer’s Disease (AD)	Aspirin 81 mg, Amlodipine/Benazepril 10 mg/20 mg, Terazosin 2 mg, Hydrochlorothiazide 25 mg, Atenolol 50 mg, Multivitamin, Calcium, Vitamin D, Atorvastatin 40 mg
Female	77	Caucasian	Hypertension (HTN), Allergic Rhinitis, Hematuria, Chronic Kidney Disease (CKD), Hypertensive Nephropathy, Hypercholesterolemia, Menopausal, Osteopenia, Gastroesophageal Reflux Disease (GERD), Large Hiatal Hernia, Gastritis, Esophagitis, Basal Cell Cancer (BCC), Degenerative Joint Disease, Rosacea, Alzheimer’s Disease (AD), Obesity, Dyspepsia	Vitamin D 2000 IU, Omeprazole 20 mg, Tylenol
Male	71	Caucasian	Atrial Fibrillation, End Stage Renal Disease (ESRD), Congestive Heart Failure (CHF), Coronary Artery Disease (CAD), Hyperlipidemia, Chronic Obstructive Pulmonary Disease (COPD), Gastroesophageal Reflux Disease (GERD), Hyperparathyroidism, Alzheimer’s Disease (AD)	Lanthanum Carbonate 1000 mg, Midodrine 10 mg, Sensipar 30 mg, Pantoprazole 40 mg, Pravastatin 40 mg, Ventolin 90 μg, Warfarin 3 mg
Female	82	Caucasian	Type 2 Diabetes, Hypothyroidism, Coronary Artery Disease (CAD), Atrial Fibrillation (AF), Hypertension (HTN), Alzheimer’s Disease (AD), Hyperlipidemia (HLD), Depression, Irritable Bowel Syndrome (IBS), Cerebrovascular Accident (CVA), Coronary Artery Disease (CAD), Vertigo, Anemia	Digoxin 125 μg, Potassium Chloride 20 mEq, Metoprolol Succinate 200 mg, Furosemide 20 mg, Levothyroxine Sodium 88 μg, Lipitor 20 mg, Memantine HCL 5 mg, Lisinopril 10 mg, Xarelto 15 mg, Amlodipine Besylate 2.5 mg, Zoloft 50 mg, Aricept 10 mg, Metformin HCL 500 mg
Male	78	Caucasian	Chronic Kidney Disease (CKD), Diabetic Nephropathy, Diabetic Neuropathy, Coronary Artery Disease (CAD), History Of Myocardial Infarction, Hyperlipidemia (HLD), Type 1 Diabetes, Depression, Age Related Macular Degeneration (AMD), Alzheimer’s Disease (AD), Dementia, Acute Renal Failure (ARF)	Humalog Mix 100 IU, Aspirin 81 mg, Centrum Silver, l-Glutamine, Metoprolol Succinate 50 mg, Lipitor 20 mg, Novolog, Humulin N, Gabapentin 100 mg, Alprazolam 0.5 mg, Fluticasone Propionate Cream, Citalopram Hydrobromide 20 mg, Cartia XT 120 mg, Aricept 5 mg, Citalopram Hydrobromide 20 mg
